# Association between Clinical Signs of Oral Lichen Planus and Oral Health-Related Quality of Life: A Preliminary Study

**DOI:** 10.3390/dj8040113

**Published:** 2020-10-04

**Authors:** Sasirin Yiemstan, Sudaduang Krisdapong, Pornpan Piboonratanakit

**Affiliations:** 1 Department of Oral Medicine, Chulalongkorn University, Bangkok 10330, Thailand; sasirin@hotmail.com; 2 Department of Community Dentistry, Chulalongkorn University, Bangkok 10330, Thailand; Sudaduang.K@chula.ac.th

**Keywords:** oral lichen planus, oral impact on daily activities, OIDP, oral health-related quality of life, thongprasom sign score

## Abstract

Subjective patient’s symptoms and Oral Health-Related Quality of Life (OHRQoL) are recommended to be involved in oral lichen planus (OLP) studies. This study aims to assess the OHRQoL of OLP patients, and their associations with pain and OLP in Thai patients. Sixty-nine patients were interviewed using the Numeric Rating Scale (NRS) for pain perception and Oral Impacts on Daily Performance (OIDP) index. OLP signs examined included localization, types, number of affected sides and clinical severity using the Thongprasom sign scoring system. There were significant associations (r_s_ = 0.490, *p* < 0.001) between clinical severity and the intensity of oral impacts as well as pain (r_s_ = 0.298, *p* = 0.013). The intensity of oral impacts and pain increased according to the increasing OLP clinical severity, except for the white striae lesions (Thongprasom sign score 1). The erosive/ulcerative OLP lesions (Thongprasom sign scores 4 and 5) were the most painful symptom and had the highest degree of oral impacts (*p* < 0.001). No significant associations were found between the number of affected lesion sides and OHRQoL (*p* = 0.316) and pain (*p* = 0.284). OHRQoL was associated with OLP type and clinical severity but not with the number of affected sides.

## 1. Introduction

Oral lichen planus (OLP) is a chronic inflammatory disease that can lead to open sores in the mouth. Most patients are female and onset occurs most commonly in the fifth or sixth decade of life [1,2,3]. The characteristics of OLP, include reticular, atrophic, erosive/ulcerative, papular and plaque types [2,4]. Many clinical indices had been established to classify OLP, and were developed, based on the clinical features, including size, color and site-based distribution. However, none of the available indices have been universally used [5]. One such system, the Thongprasom sign scoring system, has been available for categorizing the clinical severity of OLP since 1992 [6], and has been used in a number of OLP studies in many countries, including Thailand [7,8,9,10].

Common OLP symptoms vary from a burning sensation to severe chronic pain [4]. Measurement of OLP-related pain has been widely used in both clinical practice and research [11]. Despite the number of pain rating scales which exist, none are able to assess the more comprehensive and multidimensional aspects of pain [12]. Hence, Oral Health-Related Quality of Life (OHRQoL) has been suggested as it may help clinicians to thoroughly understand the pain in all aspects.

OLP is generally not life-threatening; however, the consequence of OLP could cause deterioration in OHRQoL both physical, psychological and social dimensions [13]. The impacts, such as eating difficulties with some types of food [13], which could lead to weight loss or malnutrition in severe cases, has been reported [14]. Compromised food satisfaction can affect joy and social abilities [14]. In addition, speech difficulties that could have resulted from xerostomia were also reported in OLP patients [15]. Additionally, the presence of an erosive/ulcerative lesion limits the ability to carry out daily oral hygiene practices [16]. In terms of sleeping problems, OLP patients had higher sleeping disturbances when compared with healthy persons [17], and it seems possible that lack of sleep could amplify pain signals [18], thereby increasing sensitivity to pain and might contribute to the use of the sleeping medication [17]. In focusing on the psychological disturbances, some observational studies reported greater stress and anxiety in OLP patients compared with healthy individuals [19,20]. Dissatisfaction with the appearance of OLP on the lips, including white striae, keratotic plaques, erythematous atrophic areas or ulceration, as well as the brown or black color of post-inflammatory hyperpigmentation has been reported [21,22]. This potentially affects patients’ OHRQoL because of aesthetic deterioration. In relation to the social burden, a previous study investigated the economic aspects of OLP, including social cost, work loss or school absence, which were important to the economy [23]. Lastly, the impact of OLP could cause the avoidance of social interactions, such as social gatherings or eating-out parties [13].

The concept of OHRQoL had been developed and introduced into all fields of dentistry, including oral medicine [24]. For clinicians, the application of OHRQoL revealed the importance of understanding the disease from the patient’s perspectives. Moreover, the goal of OLP treatment should focus, not only on healing the lesion and reducing pain, but also improving OHRQoL. Taking these factors into considerations, we consider that using merely clinical indicators is not sufficient, and the added value of subjective patients’ symptoms and OHRQoL in the research studies were anticipated [5,24].

Quantitative assessment of OHRQoL consists of a variety of measurement tools, both General-Health and Oral-Health quality of life indices and a specific Chronic Oral Mucosal Disease Quality of Life Index (COMDQ) [24,25]. In order to use these instruments, there is a need for cross-cultural adaption and validation of the methodology [26]. A number of previous studies have examined OHRQoL in OLP patients [27,28,29,30,31,32,33,34,35,36,37,38]. Most studies were conducted with the cross-sectional design. Various patient-based outcomes were used, for example, pain, self-perceived oral health, oral health satisfaction, as well as OHRQoL indices. Among the studies that applied the OHRQoL index, the Oral Health Impact Profile index (OHIP) was most frequently used [11,28,31,32,33,34,36,39]. The OHIP was rarely used in Thailand. The Oral Impacts on Daily Performance (OIDP) index was developed and validated in all age groups of Thai populations, namely, primary school children, adolescents, adults and elderly [40,41,42,43]. The OIDP was widely used in Thailand, including as a part of three consecutive Thailand National Oral Health surveys since 2006 until now [41,44,45]. The OHIP consists of 49 or 14 items (short form) covering a wide range of patient’s symptoms and problems of oral functioning, whereas the OIDP consists of only 8 items, which are physical, psychological and social performances in daily life. Therefore, the OIDP measures the changes in daily life performances which are considered as the ultimate oral impacts caused by various perceived symptoms [40]. To date, the OIDP has never been applied in previous studies, using Thongprasom sign scoring system.

Therefore, the aim of this study was to assess OHRQoL of OLP patients using the OIDP index. Furthermore, the associations of OHRQoL and pain perception with OLP clinical characteristics in terms of localization, type, number and severity, according to Thongprasom sign scoring system were examined.

## 2. Materials and Methods 

This cross-sectional study was approved by the Ethics Committee of the Faculty of Dentistry Chulalongkorn University (study code: HREC-DCU 2019-044, approval date of 5 July 2019), and by the Thai Clinical Trials Registry (TCTR) (study ID: TCTR 20190828002, approval date of 24 August 2019). All recruited patients received study information. Patient approval was obtained through consent forms. The study was conducted by one well-trained and calibrated interviewer between July and November 2019. The inclusion criteria were patients aged 18 or more, clinically and histopathologically diagnosed as OLP or compatible with OLP as suggested by van der Meij and van der Waal (2003) [46]. The exclusion criteria included patients with the presence of any other oral mucosal lesions, pregnancy, smokers of which other kinds of oral mucosal changes might affect quality of life and therefore, confound this study’s results. Further, emotional change during pregnancy might disturb a patient’s perception. Patients with the inability to communicate were also excluded. In terms of sample size calculation, proportions of oral impacts experienced by the patients with OLP and with aphthous lesions reported by previous similar study was used [32]. Using 85% power and 95% confidence interval level, the estimated sample size was 64. Ten percent over-sampling was applied, resulting in the total sample size of 71 patients.

Demographic characteristics were collected, included gender, age, patient types and the lesion duration since the first diagnosis of OLP, using dental records. For the clinical characteristics, OLP lesions were recorded for localization (buccal mucosa, tongue, lip, gingiva, palate, floor of the mouth and soft palate), types (reticular, atrophic, erosive/ulcerative, bullous, pigmented and plaque type), and clinical severity classified by the Thongprasom sign scoring system, demonstrated as: “0”, no lesions; “1”, white striae only; “2”, white striae with atrophic area less than 1 cm^2^; “3”, white striae with atrophic area equal to or greater than 1 cm^2^; “4”, white striae with an erosive area less than 1 cm^2^; “5”, white striae with erosive area equal to or greater than 1 cm^2^ [6]. In case of multiple OLP lesions, the highest score among all the lesions was recorded.

In relation to pain, participants were asked for the Numeric Rating Scale (NRS) pain score by stating the number that best represented their current OLP-related pain intensity, ranging from 0 to 10: “0” for no pain at all, and “10” for the worst imaginable pain. Scores were grouped into three levels of “mild pain” (0–3), “moderate pain” (4–7) and “severe pain” (8–10) [12].

OHRQoL was assessed through the Thai version of OIDP index, described elsewhere [40]. In brief, subjects were asked about whether their OLP lesions restricted their ability during the past 6 months in eight daily activities, including eating, speaking, cleaning the oral cavity, relaxing (including sleeping), emotional stability, smiling, laughing without embarrassment, carrying out major work and social contact. For each activity, frequency and severity scores were recorded. If the impacts occurred at a regular basis, the frequency was scored using 6-point scale: “0”, never affected; “1”, less than once a month; “2”, once or twice a month; “3”, once or twice a week; “4”, three to four times a week; “5”, every or nearly every day. If the impacts did not occur at a regular basis, the periodical frequency was defined by the total day of oral impacts experienced during the past 6 months in which “1”, 1–5 days; “2”, 6–15 days; “3”, 16–30 days; “4”, 1–3 months; and “5”, more than 3 months. The severity of each impacted activity on daily life was scored using a 6-point Likert scale: “0”, never affected; “1”, very little effect; “2”, little effect; “3”, moderate effect; “4”, severe effect; “5”, very severe effect [40].

To calculate the OIDP, the frequency score and the severity score were multiplied, resulting in a performance score which could range from 0–25. The sum of eight performance scores (ranging from 0–200), were divided by 2, resulting in a percentage score ranging from 0 to 100, and in which higher scores indicated poorer OHRQoL [40]. In addition to the score, we calculated “the intensity” of oral impacts which was shown to better represent the degree of subjective perception than using the percentage score [47]. The intensity of oral impact scores was allocated into five groups, based on the highest of the eight performance scores: 1–2, “very little”; 3–5, “little”; 6–12, “moderate”; 15–16, “severe”; 20–25, “very severe” [41,47]. 

### Statistical Analysis

All statistical computations were performed by SPSS statistics for Windows, version 22.0 (IBM; Armonk, NY, USA). The Kolomogonov Smirnov normality tests were applied to check for normal data distribution, however the distribution was found to be non-normal, and therefore, Mann-Whitney U tests were used. Spearman’s correlation was used to evaluate the association between the intensity of oral impacts and OLP clinical severity, pain perception (NRS) and the association between OLP pain perception and OHRQoL. The significance level was set at 5% (*p* < 0.05).

## 3. Results

### 3.1. Patient Characteristics 

A total of sixty-nine patients participated in the study (97% response rate). The study group consisted of 55 women (79.7%) and 14 men (20.3%). The mean age was 55.1 ± 13.9 years. Most participants were recall patients (82.6%). Forty-three of them (62.3%) had OLP lesions for 1–5 years; 20.3% for more than 5 years, and 17.4% less than 1 year. Almost all patients (95%) complained of having pain. However, mean pain intensities were mostly mild (59.4%), followed by moderate (34.8%) and severe (1.4%). The mean NRS pain scores were 2.56 ± 2.32.

Regarding OLP clinical characteristics, the three most common sites were buccal mucosa (88.4%), followed by gingiva (60.9%), tongue and lip (14.5%), while floor of the mouth (4.3%) and soft palate (2.9%) were infrequently affected. All patients had the reticular type of OLP. The second most common form was atrophic (95.7%), followed by erosive/ulceration (26.1%) and bullous variant (2.9%). Regarding the number of OLP affected sides, about 40% had two affected sides followed by four, and three affected sides (21.7%, 18.8%), respectively. The OLP clinical severity scores were 3 (39.1%), followed by 2 (31.9%), 4 (17.4%) and 5 (7.2%).

### 3.2. Oral Health-Related Quality of Life (OHRQoL)

The prevalence, intensity of oral impacts, percentage scores and performance scores among OLP patients are shown in Table 1. Ninety-seven percent of OLP patients had oral impacts on their daily performance. The most prevalent impacted performance was eating (88.4%) followed by cleaning the oral cavity (65.2%) and emotional stability (62.3%). In addition, there were also impacts on social activities (17.4%) and smiling (14.5%). Although the overall prevalence of oral impacts was high, the mean overall percentage score was low (12.1 ± 13.3, range 0–77.5). The highest mean performance score was that of eating (8.1 ± 6.8), followed by cleaning the oral cavity (6.6 ± 7.5) and emotional stability (5.3 ± 7.2). Regarding the intensity of oral impacts to OHRQoL, 33.3% had an impact of “moderate” intensity, followed by 18.9% with “severe” and “very severe” intensity, 13% with “very little” and “little” intensity (Table 1).

### 3.3. Association between OLP Clinical Signs and the OHRQoL and Pain Symptoms

A correlation analysis showed a statistically positive association between clinical severity and the intensity of oral impacts (r_s_ = 0.490, *p* < 0.001) (Table 2). The intensity of oral impacts increased for each step, increasing in clinical severity scores between 2 and 4. Oral impacts were perceived as little, moderate and severe to very severe intensity with clinical scores of 2, 3, and 4, respectively. Statistically significant differences in the intensity of oral impacts, compared to a one-step clinically lower score, were observed (*p* < 0.001), that is, lesions scored 2 had significantly lower oral impacts than those scored 3 (*p* = 0.002), while lesions scored 3 had significantly lower impacts than those scored 4 (*p* = 0.030). However, there was no statistically significant difference in the intensity of oral impacts between lesions scored 4 and 5 (*p* = 0.604). Moreover, patients with score 1 reported the intensity of oral impacts with severe intensity level, higher than the impacts of patients with lesions of score 2 (*p* = 0.010). 

In term of pain perception, OLP pain symptoms were significantly associated with clinical severity (r_s_ = 0.298, *p* = 0.013). Similar to oral impacts, the NRS scores continuously increased accordingly to clinical severity. The mean NRS scores rose from 1.54 ± 2.04 for score 2, to 4.00 ± 1.00 for score 5 (Table 2). Nevertheless, no statistically significant difference was observed when comparing the OLP pain perception with one-step lower clinical severity scores. In addition, more severe pain was reported in patients with score 1 (3.66 ± 1.52) than those with scores 2 and 3. The bivariate correlation between OLP pain perception and the intensity of oral impacts revealed a statistically significant positive correlation (r_s_ = 0.400, *p* = 0.001).

The OLP localization, clinical type and number of lesion were related with OHRQoL (Table 3). Our study highlighted the OLP on soft palate had a significantly greater impact on OHRQoL with very severe intensity level (*p* = 0.039), As regards the type of OLP, patients with the erosive/ulcerative OLP reported a severe to very severe intensity level, which was significantly worse than that of the other types (*p* < 0.001). Furthermore, patients with the erosive/ulcerative type of OLP had significantly higher pain, with mean NRS scores (3.88 ± 2.05), compared to the scores of the others (2.09 ± 2.24; *p* = 0.004). Additionally, neither OHRQoL nor pain perception depended on the number of affected lesion sides (*p* = 0.316, and *p* = 0.284, respectively).

## 4. Discussion

The findings from this study have extended our understanding of OLP impacts on OHRQoL. Three predominantly relevant daily activities, corresponding to a deterioration in OHRQoL, were eating, cleaning the oral cavity and emotional stability. Some participants expressed their eating behavior had been changed, in that they frequently avoided or altered some types of food and beverages thought to be causes of chronic soreness or exacerbating symptoms. These included eating softer foods with a more liquid consistency, as well as the avoidance of highly seasoned, spiced, or acidic food. Our results are similar to those of Czerninski et al.’s study, which reported that patients with tongue lesions avoided acidic citrus fruits and tomatoes [14]. In addition, our study’s participants with oral cleaning problems indicated that they had changed their oral hygiene products, such as dentifrice to the products with mild taste and smell. This finding are consistent with a previous study reporting that OLP patients were more likely to be allergic to aroma substances such as spearmint in oral hygiene products, compared to healthy subjects [16]. The participants with emotional difficulties, in our study, reported that they frequently tried to ignore or distract themselves from their problems. This finding is consistent with a previous study by Alves et al. [38] that assessed emotional state of OLP patients, compared to controlled subjects without disease. They showed that OLP patients were more likely to suffer from anxiety and depression as well as other negative impacts on quality of life. Therefore, understanding the characteristics of oral impacts caused by OLP might help clinicians give appropriate instructions to their patients. For example, modification of diet, intensive plaque control, with soft and mild oral care products, is important for patients with gingival manifestation of OLP lesions as suggested by Stone et al. [33] and Saldago et al. [37]. Furthermore, psychological support or reassurance might be required as suggested by Alves et al. [38] and Parlatescu et al. [28].

Subjective pain assessment is generally used as a patient-based outcome in OLP research. Our data exhibited a relationship between clinical severity of OLP and OHRQoL, similar to that with pain. Therefore, this finding supported the validity of the OIDP index to assess the effects of OLP on OHRQoL. The results from this study revealed, for the first time, an association between the clinical severity of OLP, according to the Thongprasom sign scoring system, and the OIDP. Greater clinical severity of OLP was associated with a poorer OHRQoL. Therefore, Thongprasom clinical scores 2 to 4 classify OLP patients according to the degree of daily life problems caused by OLP. For example, the transition from OLP clinical score 2 to score 3, reflects the enlarged atrophic area, diminished OHRQoL. Likewise, changing the clinical scores from 3 to 4 indicated that erosive/ulcerative OLP was associated with the worst OHRQoL. Contrary to expectations, the impacts of OLP with clinical severity scores of 4 and 5 on OHRQoL were unable to demonstrate a statistically significant difference, suggesting that patients might rate their ulcer’s effects to their daily activities, regardless of ulcer size. This finding agreed with that of Osipoff et al. [48]. They performed multivariate analysis and found that ulcerative size was not significantly associated with OLP symptoms. Moreover, this finding was supported by the concept proposed by Gonzalez-Moles et al. [49], noting that the intervention was considered effective if an erosive/ulcerative lesion was transformed into an atrophic lesion.

In terms of OLP types, our findings revealed that erosive/ulcerative type of OLP was associated with more painful symptom and poorer OHRQoL. This was in line with abovementioned finding, indicating OHRQoL worsening for the transition of Thongprasom clinical score 3 to score 4, and was consistent with previous studies reporting more severe pain and problems in quality of life in patients with erosive/ulcerative OLP [27,50]. Furthermore, our findings revealed that OHRQoL and pain were not significantly associated with the number of OLP lesions. This finding might be comparable with that of Osipoff et al. [48], indicating that the total area of anatomic lesion or the entire average area of generalized lesion was not significantly associated OLP symptoms.

The current finding of reticular lesions, Thongprasom sign score 1 differ from current literature that reticular lesions might not cause any or much problem to patients’ quality of life. We found that patients having such lesions reported higher impacts on OHRQoL during the past 6 months, as well as higher current pain level, than those having OLP scored at 2. Previous studies reported that patients with symptomatic reticular type were more anxious and depressed than those with non-symptomatic reticular OLP [30,31]. However, due to a very small number, namely only 3 patients having reticular OLP, further studies with bigger sample size are needed to explore OHRQoL relating to reticular OLP.

With respect to OLP pain perception, our data exhibited the relationship between OLP clinical severity and pain perception which was similar to the other OLP clinical grading criteria [51,52]. This finding confirmed the construct validity of the NRS for assessing pain perception in OLP patients. The results corroborated the earlier findings of Chainani-Wu et al. [51] who had validated the pain measurement tools in OLP patients including NRS, Visual Analogue Scale (VAS), and Change in Symptom Scale (CSS) and concluded that all three pain measuring tools were valid and reliable but NRS showed better construct validity. Furthermore, our findings revealed the superior strength of association between clinical severity and the intensity of oral impacts than NRS. This indicated a trend favoring the usefulness of the OHRQoL measurement, since it reflected the impacts not only the pain perception but also the multidimensional aspects of the life.

Our findings showed that the intensity of oral impacts and OLP pain perception did not depend upon the number of affected lesion sides, but rather on the most severe clinical lesion. As regards localization related to the OHRQoL, the OLP lesions most frequently involved were on the buccal mucosa followed by the gingiva, tongue and lip, while the involvement of the hard palate, floor of the mouth and soft palate were rarely affected. These results are in accordance with previously published studies [1,3,53]. Osipoff et al. [48] found that OLP of the tongue was the most painful lesion, which differed from our results that showed no difference in the pain perception with respect to the location. Interestingly, the present study demonstrated that OLP involving the soft palate could cause substantial impact on OHRQoL. Possible explanations for this finding were that; first, they were new patients who did not receive any treatment; and second, individuals with OLP on the soft palate reported difficulties during eating and kept worrying about the condition of their lesions, which contributed to poor OHRQoL. This might be because the soft palate is located deep in the oral cavity and has an important role in swallowing. Otherwise, the limitation of this finding was relatively small numbers of patient with soft palate involvement. More research is needed to examine more closely the association between OLP lesions at soft palate and OHRQoL impairment.

To the best of our knowledge, there has been scarcely published literature on the association between clinical severity of OLP and OHRQoL of patients, particularly OHRQoL in terms of the ability to perform daily life activities. This current study illustrates that OLP impacted patient’s daily life mostly through problems with eating, cleaning mouth and emotional state. Our findings on the associations between OLP and OHRQoL were slightly different from general expectation, that is, oral impacts were not associated with all aspects of OLP clinical characteristics. The number of lesions did not affect the degree of impacts, while the severity of each lesion did have an effect. The more clinically severe lesion, the higher impacts were perceived, with the exception of ulcerative OLP of which the impacts were highest regardless of the size of lesion. Localization of OLP also played an important role to the degree of impacts, that is, OLP at soft palate tended to cause higher impacts than those occurring at other areas. Therefore, our findings might help clinicians better understand their patients, provide treatment and instructions appropriate to their problem, as well as prioritize treatment needs according to OHRQoL.

As discussed above, these preliminary results of association analyses from current investigation were subject to certain limitations. First, our cross-sectional data would not allow for evaluating the effects of OLP treatment on OHRQoL. The data were mostly derived from follow-up patients, while 17.4% of patients were newly diagnosed who never previously been treated. For recall patients, information on OLP treatment was not available. Treatment experience in terms of type and duration of treatment might affect patient’s quality of life. Two previous longitudinal studies following OLP patients after treatments reported significantly improved clinical signs, as well as OHRQoL [33,34]. Therefore, further longitudinal study to assess overtime change of OIDP intensity, taking into account previous or ongoing treatment, would be required for better understanding on the impacts of OLP treatment on patients’ quality of life. Second, some of the previous studies performed multivariate analysis where confounding factors were taken into account [28,35]. However, this study applied only univariate analyses due to a relatively small sample size. Our small sample size led to the third limitation on the generalization of our findings to OLP patients, particularly for reticular OLP as discussed earlier. Therefore, future study with larger sample size would be required in order to corroborate the present study’s findings.

## 5. Conclusions

The current study demonstrated that nearly all patients had oral impacts affecting their daily activities. The impacts were frequently related to eating, cleaning the oral cavity and emotional stability. There were significant associations between OLP clinical signs and OHRQoL, as well as OLP pain perception among Thai OLP patients. However, some increasing clinical scores did not correspond with increasing OHRQoL. Therefore, using only an OLP sign scoring index or other clinical indicators might fail to acknowledge patient’s perceptions. The results supported the application of OHRQoL assessment to complement OLP clinical measures.

## Figures and Tables

**Table 1:** Prevalence, intensity and impact score of the Oral Impacts on Daily Performance (OIDP) in OLP patients (*n* = 69).

Total	Overall Impact	Daily Performances *n* (%)							
		Eating	Speaking	Cleaning	Relaxing Sleeping	Emotion	Smiling	Working	Social Activities
**Prevalence**	97.1	61 (88.4)	5 (7.2)	45 (65.2)	4 (5.8)	43 (62.3)	10 (14.5)	6 (8.7)	12 (17.4)
**Intensity level**									
No	2 (2.9)	8 (11.6)	64 (92.8)	24 (34.8)	65 (94.2)	26 (37.7)	59 (85.5)	63 (91.3)	57 (82.6)
Very little	9 (13)	10 (14.5)	1 (1.4)	9 (13)	0 (0)	17 (24.6)	1 (1.4)	3 (4.3)	8 (11.6)
Little	9 (13)	11 (15.9)	1 (1.4)	7 (10.1)	0 (0)	2 (2.9)	4 (5.8)	0 (0)	1 (1.4)
Moderate	23 (33.3)	23 (33.3)	2 (2.9)	11 (15.9)	0 (0)	12 (17.4)	3 (4.3)	3 (4.3)	1 (1.4)
Severe	13 (18.9)	7 (10.1)	0 (0)	12 (17.4)	1 (1.4)	5 (7.2)	0 (0)	0 (0)	1 (1.4)
Very severe	13 (18.9)	10 (14.5)	1 (1.4)	6 (8.7)	3 (4.3)	7 (10.1)	2 (2.9)	0 (0)	1 (1.4)
Impact score *									
Mean ± SD	12.1 ± 13.3	8.1 ± 6.8	0.7 ± 2.9	6.6 ± 7.5	1.2 ± 4.9	5.3 ± 7.2	1.3 ± 4.5	0.3 ± 1.3	0.9 ± 3.2
Median	8	6	0	4	0	2	0	0	0
Min–Max	0–77.5	0–25	0–20	0–25	0–25	0–25	0–25	0–6	0–20

* maximum possible score for overall impact = 100, for each performance = 25.

**Table 2:** Association and distribution of the intensity of oral impacts and NRS by the OLP clinical severity according to the Thongprasom sign score.

Thongprasom Sign Score	*n* (%)	Intensity Level (Median)	Correlation Coefficient, *p*-Value	NRS (Mean ± SD)	Correlation Coefficient, *p*-Value
1	3 (4.4)	Severe	r_s_ = 0.490 ** p < 0.001	3.66 ± 1.52	r_s_ = 0.298 * p = 0.013
2	22 (31.9)	Little ^‡^		1.54 ± 2.04	
3	27 (39.1)	Moderate ^‡‡^		2.48 ± 2.40	
4	12 (17.4)	Severe-Very severe ^‡^		3.75 ± 2.45	
5	5 (7.2)	Very severe		4.00 ± 1.00	
Total	69 (100)	Moderate		2.56 ± 2.32	

r_s_ = spearman’s correlation coefficient * correlation is significant at the 0.05 level (2-tailed), ** correlation is significant at the 0.001 level (2-tailed), ^‡^ *p* < 0.05, ^‡‡^ *p* < 0.01 (Mann-Whitney U test) compare to one-step lower clinical severity scores.

**Table 3:** Association of OLP involvement at soft palate, erosive/ulcerative OLP and number of affected lesion sides with OHRQoL and pain perception (*n* = 69).

Variables		*n* (%)	Intensity Level (Median)	*p*-Value	NRS (Mean ± SD)	*p*-Value
Soft palate	No	67 (97.1)	Moderate	*p* = 0.039 *	2.55 ± 2.35	*p* = 0.636
	Yes	2 (2.9)	Very severe		3.00 ± 0	
Erosive/ulcerative	No	51 (73.9)	Moderate	*p* < 0.001 ***	2.09 ± 2.24	*p* = 0.004 **
	Yes	18 (26.1)	Severe-Very severe		3.88 ± 2.05	
1 affected side		5 (7.2)	Moderate	*p* = 0.316	4.20 ± 3.42	*p* = 0.284
2 affected sides		28 (40.6)	Moderate		1.96 ± 2.09	
3 affected sides		13 (18.9)	Severe		2.61 ± 2.21	
4 affected sides		15 (21.7)	Moderate		3.40 ± 2.29	
5 affected sides		4 (5.8)	Very severe		2.25 ± 2.21	
6 affected sides		4 (5.8)	Moderate-severe		1.75 ± 2.36	

* *p* < 0.05, ** *p* < 0.01, *** *p* < 0.001 (Mann-Whitney U test).

## References

[1] Thongprasom K., Youngnak-Piboonratanakit P., Pongsiriwet S., Laothumthut T., Kanjanabud P., Rutchakitprakarn L. (2010). A multicenter study of oral lichen planus in Thai patients. J. Investig. Clin. Dent..

[2] Lodi G., Scully C., Carrozzo M., Griffiths M., Sugerman P.B., Thongprasom K. (2005). Current controversies in oral lichen planus: Report of an international consensus meeting. Part 1. Viral infections and etiopathogenesis. Oral Surg. Oral Med. Oral Pathol. Oral Radiol. Endod..

[3] Eisen D., Carrozzo M., Bagan Sebastian J.V., Thongprasom K. (2005). Number V Oral lichen planus: Clinical features and management. Oral Dis..

[4] Lodi G., Scully C., Carrozzo M., Griffiths M., Sugerman P.B., Thongprasom K. (2005). Current controversies in oral lichen planus: Report of an international consensus meeting. Part 2. Clinical management and malignant transformation. Oral Surg. Oral Med. Oral Pathol. Oral Radiol. Endod..

[5] Wang J., van der Waal I. (2015). Disease scoring systems for oral lichen planus; a critical appraisal. Med. Oral Patol. Oral Cir. Bucal..

[6] Thongprasom K., Luangjarmekorn L., Sererat T., Taweesap W. (1992). Relative efficacy of fluocinolone acetonide compared with triamcinolone acetonide in treatment of oral lichen planus. J. Oral Pathol. Med..

[7] Campisi G., Giandalia G., De Caro V., Di Liberto C., Aricò P., Giannola L.I. (2004). A new delivery system of clobetasol-17-propionate (lipid-loaded microspheres 0.025%) compared with a conventional formulation (lipophilic ointment in a hydrophilic phase 0.025%) in topical treatment of atrophic/erosive oral lichen planus. A phase IV, randomized, observer-blinded, parallel group clinical trial. Br. J. Dermatol..

[8] Conrotto D., Carbone M., Carrozzo M., Arduino P., Broccoletti R., Pentenero M., Gandolfo S. (2006). Ciclosporin vs. clobetasol in the topical management of atrophic and erosive oral lichen planus: A double-blind, randomized controlled trial. Br. J. Dermatol..

[9] Yoke P.C., Tin G.B., Kim M.J., Rajaseharan A., Ahmed S., Thongprasom K., Chaimusik M., Suresh S., Machin D., Bee W.H. (2006). A randomized controlled trial to compare steroid with cyclosporine for the topical treatment of oral lichen planus. Oral Surg. Oral Med. Oral Pathol. Oral Radiol. Endod..

[10] Carbone M., Arduino P.G., Carrozzo M., Caiazzo G., Broccoletti R., Conrotto D., Bezzo C., Gandolfo S. (2009). Topical clobetasol in the treatment of atrophic-erosive oral lichen planus: A randomized controlled trial to compare two preparations with different concentrations. J. Oral Pathol. Med..

[11] Wiriyakijja P., Fedele S., Porter S.R., Mercadante V., Ni Riordain R. (2018). Patient-reported outcome measures in oral lichen planus: A comprehensive review of the literature with focus on psychometric properties and interpretability. J. Oral Pathol. Med..

[12] Karcioglu O., Topacoglu H., Dikme O., Dikme O. (2018). A systematic review of the pain scales in adults: Which to use?. Am. J. Emerg. Med..

[13] Ni Riordain R., Meaney S., McCreary C. (2011). Impact of chronic oral mucosal disease on daily life: Preliminary observations from a qualitative study. Oral Dis..

[14] Czerninski R., Zadik Y., Kartin-Gabbay T., Zini A., Touger-Decker R. (2014). Dietary alterations in patients with oral vesiculoulcerative diseases. Oral Surg. Oral Med. Oral Pathol. Oral Radiol..

[15] Larsen K.R., Johansen J.D., Reibel J., Zachariae C., Rosing K., Pedersen A.M.L. (2017). Oral symptoms and salivary findings in oral lichen planus, oral lichenoid lesions and stomatitis. BMC Oral Health.

[16] Larsen K.R., Johansen J.D., Reibel J., Zachariae C., Pedersen A.M.L. (2017). Symptomatic oral lesions may be associated with contact allergy to substances in oral hygiene products. Clin. Oral Investig..

[17] Adamo D., Ruoppo E., Leuci S., Aria M., Amato M., Mignogna M.D. (2015). Sleep disturbances, anxiety and depression in patients with oral lichen planus: A case-control study. J. Eur. Acad. Dermatol. Venereol..

[18] Lumley M.A., Cohen J.L., Borszcz G.S., Cano A., Radcliffe A.M., Porter L.S., Schubiner H., Keefe F.J. (2011). Pain and emotion: A biopsychosocial review of recent research. J. Clin. Psychol..

[19] Soto Araya M., Rojas Alcayaga G., Esguep A. (2004). Association between psychological disorders and the presence of oral lichen planus, burning mouth syndrome and recurrent aphthous stomatitis. Med. Oral.

[20] Pippi R., Romeo U., Santoro M., Del Vecchio A., Scully C., Petti S. (2016). Psychological disorders and oral lichen planus: Matched case-control study and literature review. Oral Dis..

[21] Nuzzolo P., Celentano A., Bucci P., Adamo D., Ruoppo E., Leuci S., Mignogna M.D. (2016). Lichen planus of the lips: An intermediate disease between the skin and mucosa? Retrospective clinical study and review of the literature. Int. J. Dermatol..

[22] Vachiramon V., McMichael A.J. (2012). Approaches to the evaluation of lip hyperpigmentation. Int. J. Dermatol..

[23] Ni Riordain R., Christou J., Pinder D., Squires V., Hodgson T. (2016). Cost of illness of oral lichen planus in a U.K. population—A pilot study. J. Oral Pathol. Med..

[24] Ni Riordain R., Shirlaw P., Alajbeg I., Al Zamel G.Y., Fung P.L., Yuan A.D., McCreary C., Stoopler E.T., De Rossi S.S., Lodi G. (2015). World Workshop on Oral Medicine VI: Patient-reported outcome measures and oral mucosal disease: Current status and future direction. Oral Surg. Oral Med. Oral Pathol. Oral Radiol..

[25] Ni Riordain R., McCreary C. (2010). The use of quality of life measures in oral medicine: A review of the literature. Oral Dis..

[26] Berkanovic E. (1980). The effect of inadequate language translation on Hispanics’ responses to health surveys. Am. J. Public Health.

[27] Hegarty A.M., McGrath C., Hodgson T.A., Porter S.R. (2002). Patient-centred outcome measures in oral medicine: Are they valid and reliable?. Int. J. Oral Maxillofac. Surg..

[28] Parlatescu I., Tovaru M., Nicolae C.L., Sfeatcu R., Didilescu A.C. (2020). Oral health-related quality of life in different clinical forms of oral lichen planus. Clin. Oral Investig..

[29] Radwan-Oczko M., Zwyrtek E., Owczarek J.E., Szcześniak D. (2018). Psychopathological profile and quality of life of patients with oral lichen planus. J. Appl. Oral Sci..

[30] Adamo D., Cascone M., Celentano A., Ruoppo E., Leuci S., Aria M., Mignogna M.D. (2017). Psychological profiles in patients with symptomatic reticular forms of oral lichen planus: A prospective cohort study. J. Oral Pathol. Med..

[31] Vilar-Villanueva M., Gandara-Vila P., Blanco-Aguilera E., Otero-Rey E.M., Rodriguez-Lado L., Garcia-Garcia A., Blanco-Carrion A. (2019). Psychological disorders and quality of life in oral lichen planus patients and a control group. Oral Dis..

[32] Tabolli S., Bergamo F., Alessandroni L., Di Pietro C., Sampogna F., Abeni D. (2009). Quality of life and psychological problems of patients with oral mucosal disease in dermatological practice. Dermatology.

[33] Stone S.J., Heasman P.A., Staines K.S., McCracken G.I. (2015). The impact of structured plaque control for patients with gingival manifestations of oral lichen planus: A randomized controlled study. J. Clin. Periodontol..

[34] McGrath C., Hegarty A.M., Hodgson T.A., Porter S.R. (2003). Patient-centred outcome measures for oral mucosal disease are sensitive to treatment. Int. J. Oral Maxillofac. Surg..

[35] Aleksejūnienė J., Rimkevicius A., Puriene A., Rasteniene R. (2019). Self-perceived oral health, satisfaction with overall health and quality of life comparisons between patients with oral lichen planus and their matched controls. J. Contemp. Dent..

[36] López-Jornet P., Camacho-Alonso F. (2010). Quality of life in patients with oral lichen planus. J. Eval. Clin. Pract..

[37] Salgado D.S., Jeremias F., Capela M.V., Onofre M.A., Massucato E.M., Orrico S.R. (2013). Plaque control improves the painful symptoms of oral lichen planus gingival lesions. A short-term study. J. Oral Pathol. Med..

[38] Alves M.G., do Carmo Carvalho B.F., Balducci I., Cabral L.A., Nicodemo D., Almeida J.D. (2015). Emotional assessment of patients with oral lichen planus. Int. J. Dermatol..

[39] Zucoloto M.L., Shibakura M.E.W., Pavanin J.V., Garcia F.T., da Silva Santos P.S., Maciel A.P., de Barros Gallo C., Souza N.V., Innocentini L., Humberto J.S.M. (2019). Severity of oral lichen planus and oral lichenoid lesions is associated with anxiety. Clin. Oral Investig..

[40] Adulyanon S., Sheiham A., Slade G.D. (1997). Oral impacts on daily performances. Measuring Oral Health and Quality of Life.

[41] Krisdapong S., Sheiham A., Tsakos G. (2009). Oral health-related quality of life of 12- and 15-year-old Thai children: Findings from a national survey. Community Dent. Oral Epidemiol..

[42] Gherunpong S., Tsakos G., Sheiham A. (2004). Developing and evaluating an oral health-related quality of life index for children; the child-OIDP. Community Dent. Health.

[43] Srisilapanan P., Korwanich N., Sheiham A. (2003). Assessing prosthodontic dental treatment needs in older adults in Thailand: Normative vs. sociodental approaches. Spec. Care Dentist..

[44] Prasertsom P., Kaewkamnerdpong I., Krisdapong S. (2020). Condition-specific oral health impacts in Thai children and adolescents: Findings from the national oral health-related quality of life survey. Asia Pac. J. Public Health.

[45] Dental Health Division, Health Division (2017). Report on the Eighth Thailand National Oral Health Survey.

[46] Van der Meij E.H., van der Waal I. (2003). Lack of clinicopathologic correlation in the diagnosis of oral lichen planus based on the presently available diagnostic criteria and suggestions for modifications. J. Oral Pathol. Med..

[47] Krisdapong S., Sheiham A. (2014). Which aspects of an oral health-related quality of life measure are mainly associated with global ratings of oral health in children?. Community Dent. Oral Epidemiol..

[48] Osipoff A., Carpenter M.D., Noll J.L., Valdez J.A., Gormsen M., Brennan M.T. (2020). Predictors of symptomatic oral lichen planus. Oral Surg. Oral Med. Oral Pathol. Oral Radiol..

[49] Gonzalez-Moles M.A., Bravo M., Gonzalez-Ruiz L., Ramos P., Gil-Montoya J.A. (2018). Outcomes of oral lichen planus and oral lichenoid lesions treated with topical corticosteroid. Oral Dis..

[50] Suliman N.M., Johannessen A.C., Ali R.W., Salman H., Astrom A.N. (2012). Influence of oral mucosal lesions and oral symptoms on oral health related quality of life in dermatological patients: A cross sectional study in Sudan. BMC Oral Health.

[51] Chainani-Wu N., Silverman S., Reingold A., Bostrom A., Lozada-Nur F., Weintraub J. (2008). Validation of instruments to measure the symptoms and signs of oral lichen planus. Oral Surg. Oral Med. Oral Pathol. Oral Radiol. Endod..

[52] Park H.K., Hurwitz S., Woo S.B. (2012). Oral lichen planus: REU scoring system correlates with pain. Oral Surg. Oral Med. Oral Pathol. Oral Radiol..

[53] Kaomongkolgit R., Daroonpan P., Tantanapornkul W., Palasuk J. (2019). Clinical profile of 102 patients with oral lichen planus in Thailand. J. Clin. Exp. Dent..

